# Directivity enhancement of a cylindrical wire antenna by a graded index dielectric shell designed using strictly conformal transformation optics

**DOI:** 10.1038/s41598-021-92200-4

**Published:** 2021-06-22

**Authors:** Hossein Eskandari, Soorena Saviz, Tomáš Tyc

**Affiliations:** 1 Department of Electrical Engineering, Ferdowsi University of Mashhad, Mashhad, Iran; 2 Department of Theoretical Physics and Astrophysics, Faculty of Science, Masaryk University, Kotlářská 2, 61137 Brno, Czechia

**Keywords:** Transformation optics, Electrical and electronic engineering

## Abstract

A transformation-optical method is presented to enhance the directivity of a cylindrical wire antenna by using an all-dielectric graded index medium. The strictly conformal mapping between two doubly connected virtual and physical domains is established numerically. Multiple directive beams are produced, providing directive emission. The state-of-the-art optical path rescaling method is employed to mitigate the superluminal regions. The resulting transformation medium is all-dielectric and nondispersive, which can provide broadband functionality and facilitate the realization of the device using available fabrication technologies. The realization of the device is demonstrated by dielectric perforation based on the effective medium theory. The device’s functionality is verified by carrying out both ray-tracing and full-wave simulations using finite-element-based software COMSOL Multiphysics.

## Introduction

Finding a medium that performs the desired functionality is essentially an inverse problem. This problem becomes more challenging to solve as the desired functionality becomes more sophisticated. Transformation optics (TO) provides a systematic approach to finding a material that provides specific functionality by establishing a geometrical mapping between two spaces, namely virtual and physical spaces. The propagation characteristics of an electromagnetic wave depend not only on the material but also on the geometry of the space. Based on the form-invariance of Maxwell’s equations, TO implies that a space deformation can be embodied in a transformation medium. The cloak of invisibility was the first and the most well-known application of TO^1,2^ where an engineered material guides the electromagnetic wave around a hidden object. Inspired by the invisibility cloak idea, researchers began to adapt TO to various applications, providing interesting functionalities like carpet cloaking (also known as plasmonic bump cloaking)^3–9^, polarization splitting and transforming^10–14^, directivity enhancing^15–18,18–21^, beam expanding^22,23^, waveguide coupling^24–30^, and lens compression^31–34^.

Despite the fascinating applications of TO, the derived transformation materials are often complex, demanding considerable anisotropy and spatial dependence. This complexity depends mainly on two factors, the expected functionality and the type of mapping employed. For a specific application, a general form of transformation often leads to a nonhomogeneous and anisotropic material, which demands both electric and magnetic constitutive parameters. Practically speaking, meeting all these requirements is impossible. Restricting the affected polarization can remove the magnetic medium requirement in some cases^12,14,26,27,35–37^. Besides, certain classes of transformations can alleviate some of the material complexities. For instance, using a linear transformation leads to a homogeneous material. Also, an exciting case that finds many practical applications is when conformal (CTO) or quasi-conformal transformation optics (QCTO) are used to manipulate the TE polarization (out-of-plane electric field component). The resultant material, in this case, is a graded index (GRIN) isotropic nonhomogeneous dielectric^38^. Having such GRIN material opens up a wide range of fabrication methods to realize the device, including drilling sub-wavelength holes in a dielectric substrate^5,39,40^, using graded photonic crystals^41–43^, dielectric layered shell deposition^39,44,45^, electron-beam lithography^46–48^, and 3D printing^49,50^. Owing to their practicality, CTO and QCTO have drawn a great deal of attention.

CTO and QCTO have been successfully applied to the directivity enhancement applications^15–21^. Following a numerical conformal method, a GRIN lens embedded inside the body of a horn was proposed to mitigate the phase error at the antenna aperture^17,18^. Also, using CTO and QCTO, a transformation medium was proposed to produce a single directive beam from a cylindrically radiating point source^16,21^. A closed-form formula was presented for the conformal mapping between a circle and a square^15^. This could create four directive beams from a point source located at the center of the physical space. Following a similar idea but using a different tool, a numerically calculated QCTO design was put forward to achieve the same goal^19,20^.

Usually, biconical, discone, dipole, and monopole antennas are employed to provide an omnidirectional pattern^51^. It may be necessary to implement a wire antenna with a larger radius to enhance the bandwidth or make it mechanically more robust. In addition, shrinking the GRIN medium around the structure to make the design more compact reduces the relative size of the transformation medium. As a result, the point source assumption becomes inaccurate both mathematically (based on the transformation optics basis) and physically. Having a cylindrical wire antenna with a non-zero radius as the source reflects the reality better. A multi-beam transformation optical solution for the directivity enhancement of a cylindrical wire antenna has not yet been proposed in the literature. In contrary with the case of a point source where the transformation is established between two simply connected regions, for the case of a wire, the conformal mapping needs to be established between two doubly connected regions.

Here, a strictly conformal transformation is derived that produces several directive beams from a cylindrical wire antenna with a non-zero radius. The calculated GRIN medium gradually flattens the cylindrical phase fronts of the wire antenna. The conformal mapping between the two doubly connected virtual and physical spaces is calculated numerically. Similar to other works in the literature, TO inherently creates regions with a superluminal refractive index. The optical path rescaling is used to control the refractive index. A prototype of the design is realized by perforating a dielectric substrate based on the effective medium theory. Finite-element-based ray-tracing and full-wave simulations confirm the functionality of the proposed design method.

## Design method

### Transformation optics and conformal mapping

TO establishes a relation between the material and the electromagnetic fields between two spaces, namely virtual space and physical space. Here, the Cartesian coordinates for the virtual space (*w*-plane) and physical space (*z*-plane) are declared as (*u*, *v*) and (*x*, *y*) for the 2D case. If the complex variables $w=u+{\mathrm{i}}v$ and $z=x+{\mathrm{i}}y$ are defined, the conformal mapping from the physical space to the virtual space can be expressed as an analytic function $w=f(z)$. In that case, if the material in the virtual space is taken as vacuum, CTO implies that the refractive index of the GRIN transformation medium in the physical space follows the below equation^2^:

1 $$\begin{aligned} n(x,y) = \left| {\frac{{\mathrm{{d}}f(z)}}{{\mathrm{{d}}z}}} \right| {} = \left| {f'(z)} \right|. \end{aligned}$$

It is worth mentioning that the above refractive index is derived based on the assumption that TE polarization is being affected by the transformation. This assumption is accurate for the case of a cylindrical wire with an electrical current along the *z*-axis. Once the conformal mapping $w=f(z)$ is calculated, a simple differentiation yields the refractive index, which is clearly dependent on the physical space coordinates, hence making the material nonhomogeneous.

### Enhancing the directivity of a wire antenna using CTO

**Figure 1:**
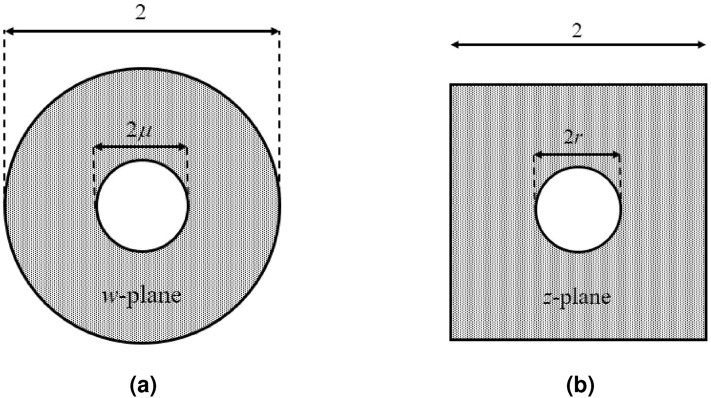
Schematic of the virtual and physical spaces for the directivity enhancement of a cylindrical wire antenna. (**a**) Virtual space, and (**b**) physical space with a square outer boundary.

Figure 1 depicts the virtual and physical spaces for enhancing the directivity of a cylindrical wire. The virtual space is an annulus with an inner radius of $\mu$ and an outer radius of 1. On the other hand, the physical space is a doubly connected shape where a square (as a special case) encloses the cylindrical wire with the radius of *r*.

The phase fronts of the wave radiating from a wire have cylindrical contours. Due to the conformal mapping, these cylindrical contours are expected to gradually transform into square contours as we move from the source to the outer polygon. Hence the final transformation medium will gradually flatten the cylindrical phase fronts of the wave emanating from the cylindrical wire. Such conformal transformation involves mapping two doubly connected regions.

An interesting theorem^52^ implies that “let *D* be a nondegenerate doubly connected region, then there exists a unique real number $\mu$, $0< \mu < 1$, such that there exists a one-to-one analytic function *f*(*z*) that maps *D* onto the annulus $R_\mu$: $\mu< \left| w \right| < 1$. If the outer boundaries correspond to each other, then *f*(*z*) is determined up to a rotation of the annulus”. The unique value ${\mu ^{ - 1}}$ is called the conformal modulus of *D*. This means that for a given doubly connected physical space, there exists a unique annulus with a specified inner radius of $\mu$ and outer radius of 1. Note that the only degree of freedom is a rotation, which is not beneficial to our application.

**Figure 2:**
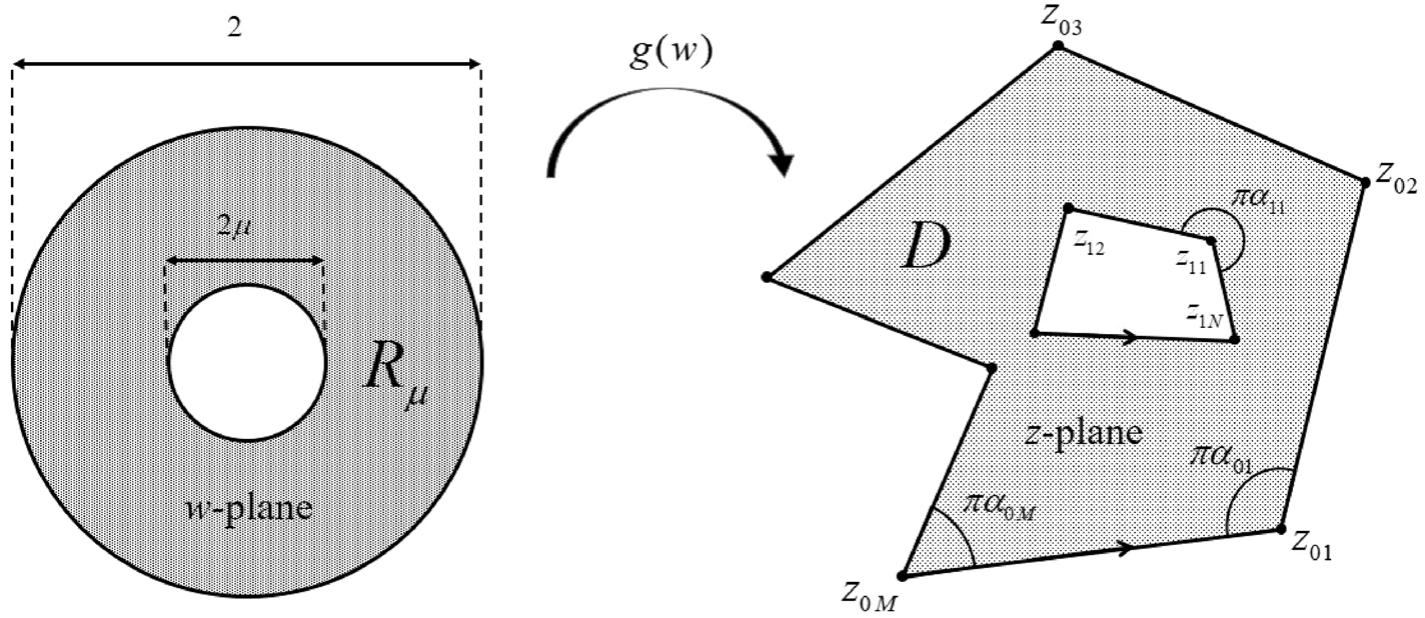
The illustration of the geometries associated with the conformal mapping from the annulus $R_\mu$ to the doubly connected polygon *D*.

Figure 2 illustrates the geometries for the conformal mapping of the annulus $R_\mu$ to the doubly connected polygon *D*.

It is more favorable to calculate the conformal mapping *g*(*w*) from the annulus $R_\mu$ to the doubly connected polygon *D*. For such a case, the mapping can be expressed based on the closed-form Schwarz-Christoffel formula^53–55^:

2 $$\begin{aligned} g(w) = g({w_c}) + C\int _{{w_c}}^w {\prod \limits _{k = 1}^M {{{\left[ {\theta \left( {\frac{w}{{\mu {w_{0k}}}}} \right) } \right] }^{{\alpha _{0k}} - 1}}{{\prod \limits _{k = 1}^N {\left[ {\theta \left( {\frac{{\mu w}}{{{w_{1k}}}}} \right) } \right] } }^{{\alpha _{1k}} - 1}}dw} }. \end{aligned}$$

where $\left\{ {{w_{0k}}} \right\} _{k = 1}^M$ and $\left\{ {{w_{1k}}} \right\} _{k = 1}^N$ are the points located on the inner and outer rims of the annulus $R_\mu$, respectively. These set of points are the prevertices of the vertex points $\left\{ {{z_{0k}}} \right\} _{k = 1}^M$ and $\left\{ {{z_{1k}}} \right\} _{k = 1}^N$ located on the inner and outer contours of polygon *D* in a counterclockwise order. *C* is a complex constant and ${w_c} \in {R_u}$. $\left\{ {\pi {\alpha _{0k}}} \right\} _{k = 1}^M$ and $\left\{ {\pi {\alpha _{1k}}} \right\} _{k = 1}^N$ are the polygon’s internal angles. Also, the $\theta$ function follows the below equation^55^:

3 $$\begin{aligned} \theta (w) = \prod \limits _{d = 0}^\infty {\left( {1 - {\mu ^{2d + 1}}w} \right) \left( {1 - {\mu ^{2d + 1}}{w^{ - 1}}} \right) .} \end{aligned}$$

The calculation of all the accessory parameters $\left\{ {{w_{0k}}} \right\} _{k = 1}^M$, $\left\{ {{w_{1k}}} \right\} _{k = 1}^N$, constant *C*, and the inner radius $\mu$ is handled numerically.

Here, we select three shapes for the outer boundary of the physical space. An equilateral triangle with the side length of 2, a square with the side length of 2, and a hexagon with the side length of 1. The inner boundary of all three cases is a circle with a radius of $r=0.2$ m. Following the numerical method, the conformal module ${\mu ^{ - 1}}$ for the three cases is derived, and it equals 2.83, 5.40, and 4.49, respectively. This implies that each of the three physical space shapes is conformally equivalent to an annuluses $R_\mu$ with an outer radius of 1 and an inner radius $\mu$ of 0.35, 0.185, and 0.22 m, respectively.

**Figure 3:**
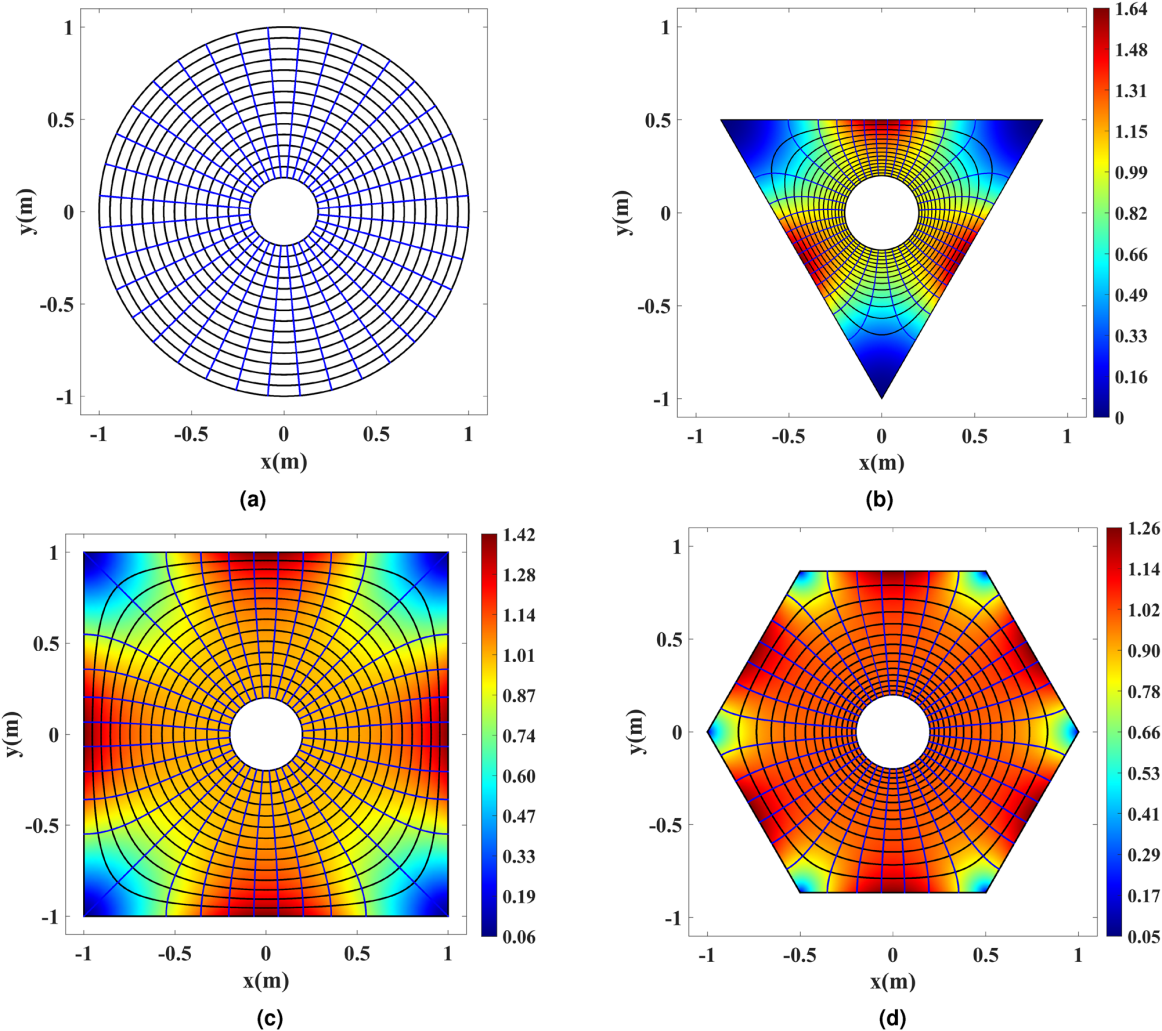
Grid transformation between the virtual and physical spaces. The solid blue lines represent the rays and the solid black lines represent the phase fronts. (**a**) Cylindrical grids in the virtual space annulus. The refractive index and the mapped grids inside (**b**) the triangular, (**c**) the square, and (**d**) the hexagonal physical spaces.

The conformal mapping function *g*(*w*) from the virtual space to the physical space’s polygon is calculated for all three cases numerically. Figure 3 depicts the transformation of cylindrical grids from the virtual to the physical space polygons. The solid blue radial lines and solid black cylindrical contours in Fig. 3a represent the rays’ propagation direction and phase front contours. Based on Fig. 3, it is seen that the calculated conformal map flattens the phase front contours and keeps the rays’ direction perpendicular to the outer shell boundaries in the physical space. Hence, the device is expected to produce multiple directive in-phase beams. The refractive index is illustrated in Fig. 3b–d. The final refractive index is multiplied to $r/\mu$ to change the refractive index near the inner circle to 1. It is worth mentioning that applying any multiplication to the refractive index is equivalent to adding a geometry scaling, which is also a conformal map. Our conformal mapping transforms the inner rim of the virtual space with the radius of $\mu$ to the one of the physical space with the radius of *r*, which is basically a scaling. Multiplying the index to $r/\mu$ cancels this scaling off and leads to a unity transformation and hence a unity refractive index near the inner rim.

As can be seen from Fig. 3, the unique conformal map *g*(*w*) intrinsically exerts space expansion to the annulus near the physical space’s corners. This creates refractive index values below one. Also, the maximum required refractive index decreases as the number of polygon sides increases. For instance, the hexagon requires more moderate refractive index values. The physical space and the virtual space will become more similar as the number of sides increases. CTO leads to a unity transformation and a unity refractive index as the number of polygon sides goes to infinity. We select the hexagon shape here. One requires dispersive narrowband metamaterial structures to realize the superluminal refractive index regions. Although there is no way to remedy this defect fully, we can significantly reduce it by employing the optical path rescaling method^9^, bringing the refractive index to a very moderate range.

### Controlling the refractive index by optical path rescaling

**Figure 4:**
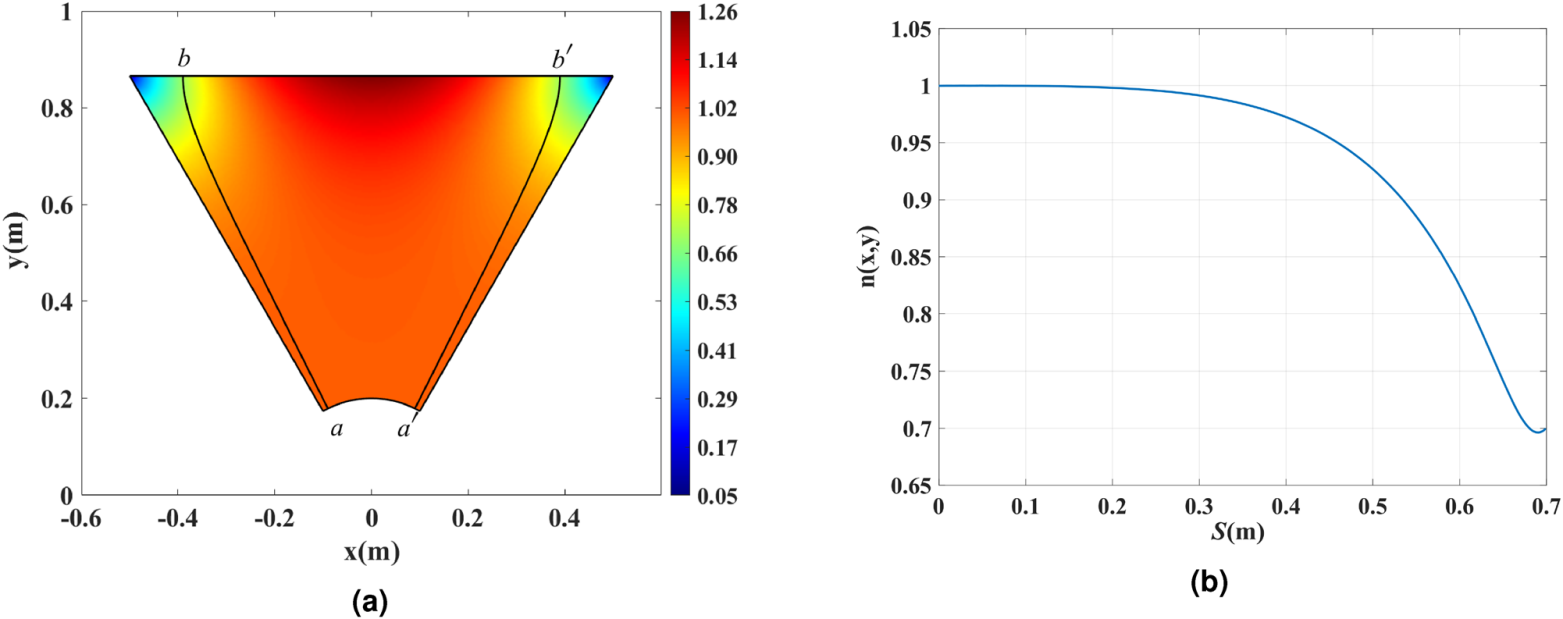
(**a**) The upper $\pi /3$ radians sector of Fig. 3d hexagon and its refractive index. The ray *ab* is shown together with its mirror-symmetric ray $a^{\prime }b^{\prime }$. (**b**) The refractive index versus the optical path length along the ray *ab*.

We describe the optical path rescaling method using a single sector of the hexagon; the procedure is then applied analogously to all six sectors. Figure 4a depicts the upper sector of $\pi /3$ radians of Fig. 3d. Consider the rays *ab* and $a'b'$ shown by the solid black lines in Fig. 4b that are mirror-symmetric with respect to one another. Ray *ab* corresponds, in physical space, to the radial line in the virtual space of Fig. 3a at the $\phi = {117^ \circ }$ angle; this value is chosen to eliminate most of the superluminal region while keeping the resulting refractive index profile at moderate levels. We aim to control the refractive index inside $aa'b'ba$ region. The ray *ab* touches the upper side of the sector at the point *b* with coordinates $(-0.39\,{\mathrm{m}}, \sqrt{3}/2\,{\mathrm{m}})$. We can define an optical path associated with any point A lying on ray *ab* as an integral

4 $$\begin{aligned} S({\mathrm{A}}) = \int _a^{\mathrm{A}} n\,{\mathrm{d}} l\,. \end{aligned}$$

Here the integral is taken along the ray from the point *a* (where we set the optical path to zero) to the point A. The optical path defined in this way is proportional to the phase of the wave associated with the rays with a proportionality factor of $1/k=c/\omega$. Now, we can define the optical path *S*(*x*, *y*) in the whole sector similarly: since any point ${\mathrm{B}}=(x,y)$ of the sector lies on exactly one ray (according to our design of the rays in Fig. 3d), we calculate the function *S*(*x*, *y*) in analogy to Eq. (4) as an integral of the refractive index along that ray from a point $a''$ to B, where $a''$ is the point on the intersection of that ray with the arc $aa'$. The refractive index then satisfies $n(x,y)=|\nabla S(x,y)|$, and the direction of $\nabla S(x,y)$ is tangent to the ray passing through the given point.

Now, we take advantage of the fact that there may be different refractive index distributions that produce the same set of rays. In other words, the same set of rays of Fig. 3d can be produced not just by the index profile *n*(*x*, *y*) but also by an alternative profile $n'(x,y)$. To find it, we define an increasing function $S'(S)$ that we call the “rescaled optical path”. If we imagine that $S'$ rather than *S* describes the optical path, then the corresponding refractive index will be

5 $$\begin{aligned} n'(x,y) = |\nabla S'|=|\nabla S|\,\frac{{\mathrm{d}}S'}{{\mathrm{d}}S}=n\,\frac{{\mathrm{d}}S'}{{\mathrm{d}}S}. \end{aligned}$$

Since the direction of the vector $\nabla S'$ is the same as the direction of $\nabla S$, the direction of the ray passing through any point B has not changed upon the rescaling; this shows that the set of rays corresponding to the rescaled optical path $S'(x,y)$ (and the index $n'(x,y)$) is indeed the same as the set of rays corresponding to the optical path *S*(*x*, *y*) (and the index *n*(*x*, *y*)).

We can now employ the freedom contained in the function $S'(S)$ to manipulate the index according to our needs. In particular, we will use it to eliminate the superluminal index inside the $aa'b'ba$ region in Fig. 4a. To do that, we require the rescaled index $n'$ on ray *ab* to be unity; the original refractive index *n* along this ray is plotted versus the optical path in Fig. 4b.

**Figure 5:**
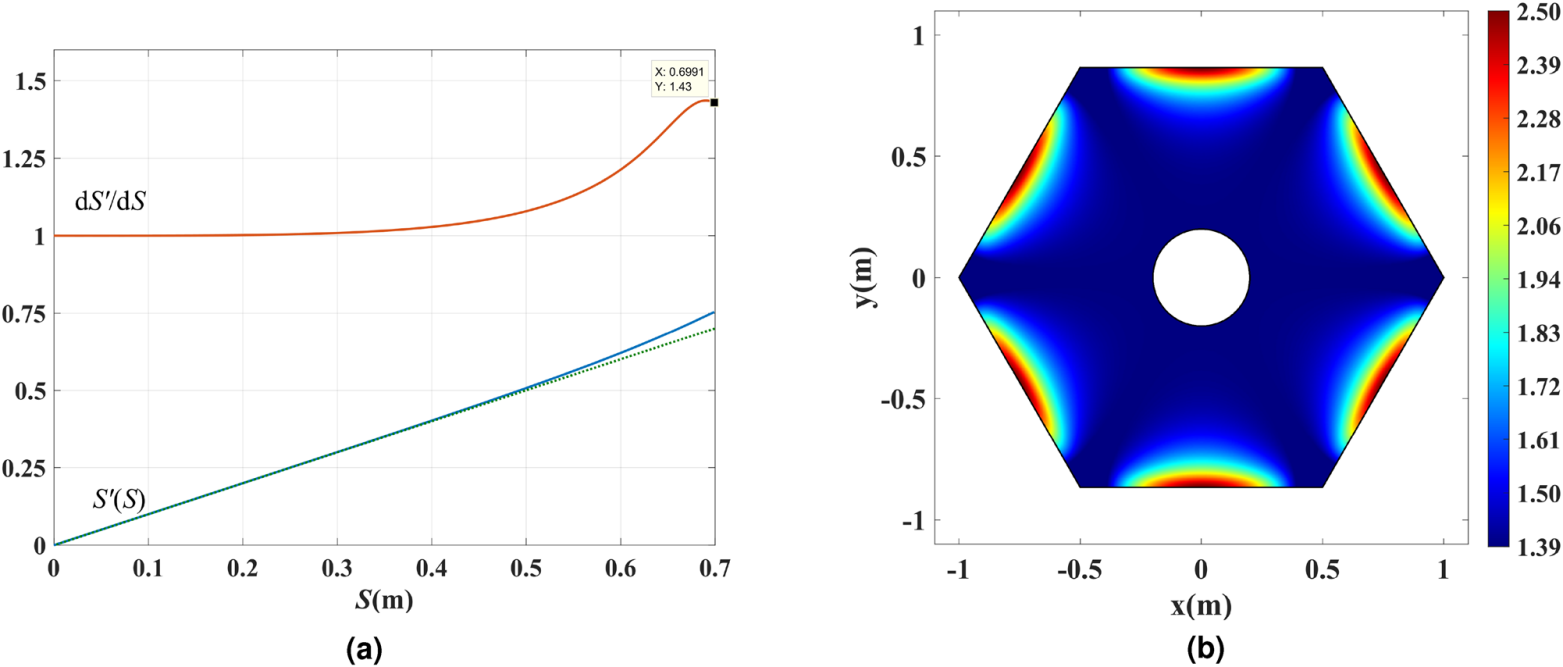
(**a**) The scaling function $S'(S)$ and its derivative ${\mathrm{{d}}}S'/{\mathrm{{d}}}S$. (**b**) The refractive index of the hexagonal physical space in Fig. 3d after rescaling the optical path for mitigating the superluminal regions and applying index multiplication.

Since on the ray *ab* we require $n'=|\nabla S'|=1$, Eq. (5) yields ${\mathrm{d}}S'/{\mathrm{d}}S=1/n$, which leads to the following scaling function:

6 $$\begin{aligned} S'(S) = \int \limits _0^S {\frac{1}{{n(S)}}{} {\mathrm{{d}}}S} \end{aligned}$$

defined on the interval $S\in [0,0.7]$ that corresponds to the optical path along the ray *ab*. The function $S'(S)$ fully defines the new refractive index profile $n'(x,y)$ in the whole sector via Eq. (5). Figure 5a shows the function $S'(S)$ and its derivative ${\mathrm{d}}S'/{\mathrm{d}}S$.

It is worth mentioning that any other ray could be chosen as the candidate ray for employing the optical path rescaling. Since the $aa'b'ba$ region is equivalent to the region sandwiched between $\phi = {117^ \circ }$ and $\phi = {63^ \circ }$ in the virtual space, we expect to utilize a total of ${54^ \circ }$ out of ${60^ \circ }$ (90% of the power) by choosing the ray *ab* as the rescaling reference. Choosing a ray with endpoints closer to the corners will provide us with a larger aperture but will lead to very large refractive index values. After applying the rescaling, the index inside the $aa'b'ba$ region will rise above unity while having the unity refractive index on boundaries corresponding to *ab* and $a'b'$ rays. The regions outside the $aa'b'ba$ area will be replaced by the minimum available refractive index and are not expected to contribute to the directivity enhancement.

Since the value of ${\mathrm{{d}}}S'/{\mathrm{{d}}}S=1.43$ at $S=0.7$, it is expected for the maximum refractive index to reach $n' = 1.26\times 1.43=1.8$ near the middle of the top boundary in Fig. 4a. We aim to use Arlon AD600 substrate, which provides the maximum refractive index of 2.51 with the loss tangent of $\tan \delta = 0.0029$. As shown in the next subsection, using AD600 and employing substrate perforation, one can attain refractive index values in the range of $1.4 \le n' \le 2.51$. Since the refractive index of regions outside the rescaling region will be replaced by $n'=1.4$, we multiply the refractive index of the device to 1.4. The final refractive index will fall within the achievable refractive index of a perforated Arlon AD600. The refractive index of the hexagonal dielectric shell after applying the rescaling and index multiplication is shown in Fig. 5b for the whole physical space, i.e., in all the six rescaled segments combined.

### Realization of the refractive index by using the effective medium theory

The realization of the refractive index in Fig. 5b is done using sub-wavelength hexagonal unit cells and based on the effective medium theory. The hexagonal lattice can form a regular tessellation of the 2D space while providing a high filling factor, which is favorable for achieving lower minimum effective refractive index values. The square unit cell can form a regular tessellation too but provides lower filling factor values and a higher minimum effective refractive index. Using the hexagonal unit cell provides us with a reasonable, achievable refractive index range and a robust tessellation of the space.

**Figure 6:**
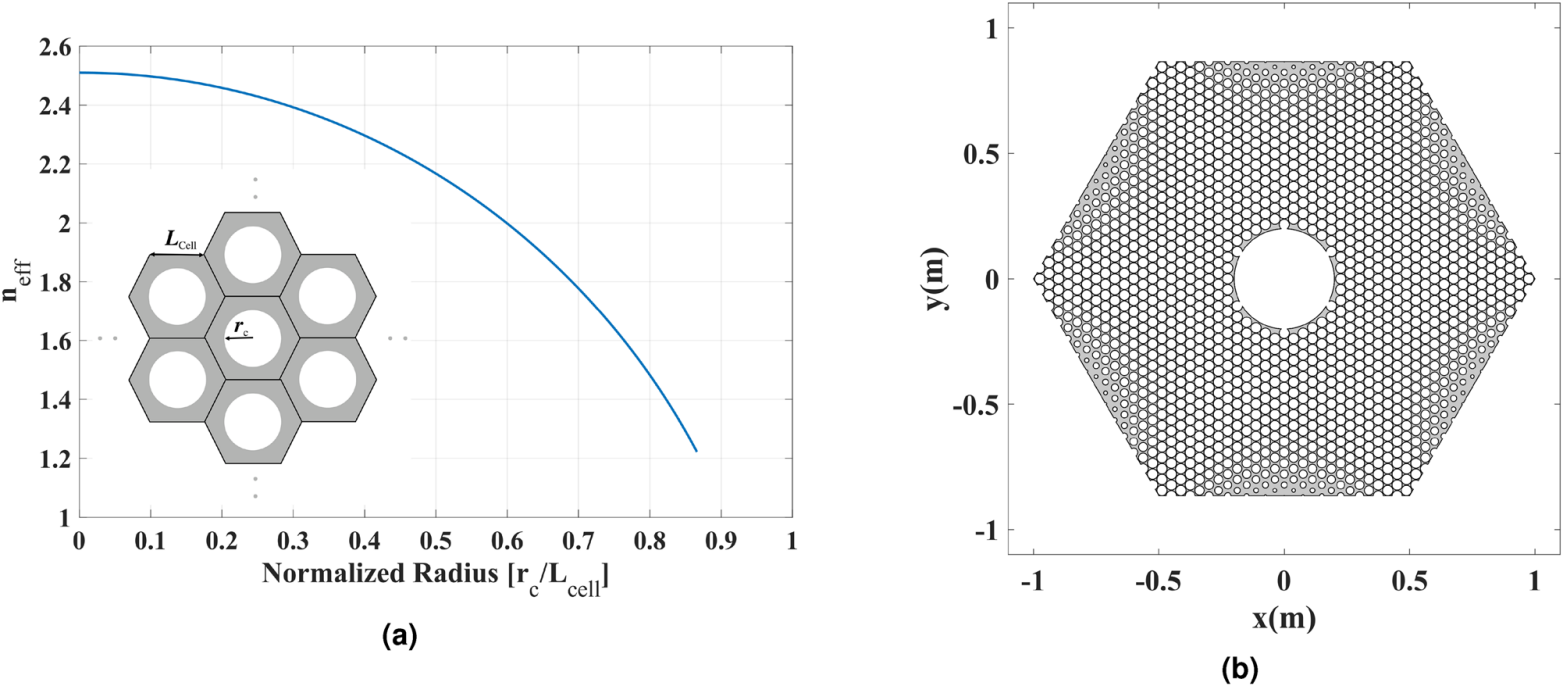
(**a**) The effective refractive index of a hexagonal unit cell versus ${r_{\mathrm{{c}}}}$ normalized to ${L_{\mathrm{{cell}}}}$. The hexagonal lattice is illustrated in the subset. (**b**) The perforated Arlon AD600 that mimics the refractive index of Fig. 5b.

Here, we employed air-filled cylindrical perforations inside the AD600 host material with the relative permittivity of ${\varepsilon _{\mathrm{{host}}}} = 6.3$. The arrangement for such structure is depicted in the subset of Fig. 6a. We can control the effective refractive index of each cell by changing the cylindrical perforation radius. The unit cell’s effective relative permittivity ${\varepsilon _{\mathrm{{eff}}}}$ for TE polarization follows:

7 $$\begin{aligned} {\varepsilon _{\mathrm{{eff}}}} = {f_{\mathrm{{cell}}}} + \left( {1 - {f_{\mathrm{{cell}}}}} \right) {\varepsilon _{\mathrm{{host}}}}, \end{aligned}$$

where ${f_{\mathrm{{cell}}}} = 2\pi r_{\mathrm{{c}}}^2/\left( {3\sqrt{3} L_{\mathrm{{cell}}}^2} \right)$ is the filling factor of a hexagonal unit cell, ${r_{\mathrm{{c}}}}$ is the radius of the perforated cylinder, and ${L_{\mathrm{{cell}}}}$ is the hexagonal cell side length. The effective refractive index of the hexagonal unit cell is plotted in Fig. 6a versus ${r_{\mathrm{{c}}}}$ normalized to ${L_{\mathrm{{cell}}}}$.

The hexagonal unit cell can provide a filling factor of up to $\pi /2\sqrt{3} \approx 0.91$ (for ${r_{\mathrm{{c}}}} = \sqrt{3} {L_{\mathrm{{cell}}}}/2$), and the minimum effective refractive index of 1.22 theoretically. However, in such a case, the cylindrical hole touches the body of the hexagonal cell, which is mechanically undesirable. One should take smaller filling factors to ensure mechanical stability.

Having the required relative permittivity based on the refractive index distribution of the physical space in Fig. 5b, the radius of the perforated cylinder ${r_{\mathrm{{c}}}}$ inside the hexagonal unit cells is given by:

8 $$\begin{aligned} {r_{\mathrm{{c}}}} = {L_{\mathrm{{cell}}}}\sqrt{\frac{{3\sqrt{3} \left( {{\varepsilon _{\mathrm{{host}}}} - {\varepsilon _{\mathrm{{eff}}}}} \right) }}{{2\pi \left( {{\varepsilon _{\mathrm{{host}}}} - 1} \right) }}} . \end{aligned}$$

﻿Based on Eqs. (7) and (8), the perforated AD600 with the filing factor values of $0.01 \le {f_{\mathrm{{cell}}}} \le 0.82$ can mimic the refractive index range of $1.4 \le n' \le 2.5$ in Fig. 5b. The perforated structure is shown in Fig. 6b.

## Simulation results

**Figure 7:**
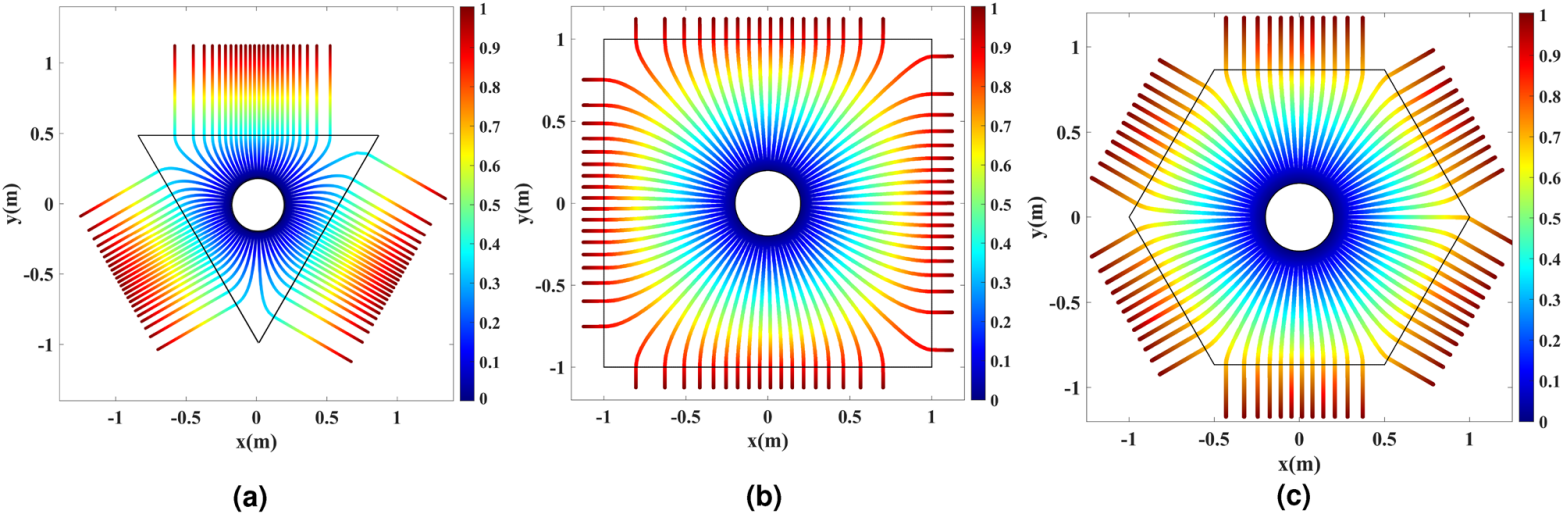
The ray-tracing results for 73 equally-spaced rays launched from the physical space’s inner rim. The results are shown for the transformation medium depicted in (**a**) Fig. 3b, (**b**) Fig. 3c, and (**c**) Fig. 3d. The color expression indicates the optical path length.

Full-wave and ray-tracing simulations are carried out to verify the functionality of the designs. The first simulation is ray-tracing, which is based on geometrical optics (GO) and verifies the element’s high-frequency behavior. The simulation is done for the triangular, square, and hexagonal dielectric shells in Fig. 3b–d. To excite the inner rim of the physical space, 73 equally-spaced rays are launched from the rim’s circumference. Figure 7 illustrates the ray trajectories with the color expression of the optical path length. The directivity enhancement is seen as the rays exit the physical space polygon perpendicular to the sides with the same optical path length.

**Figure 8:**
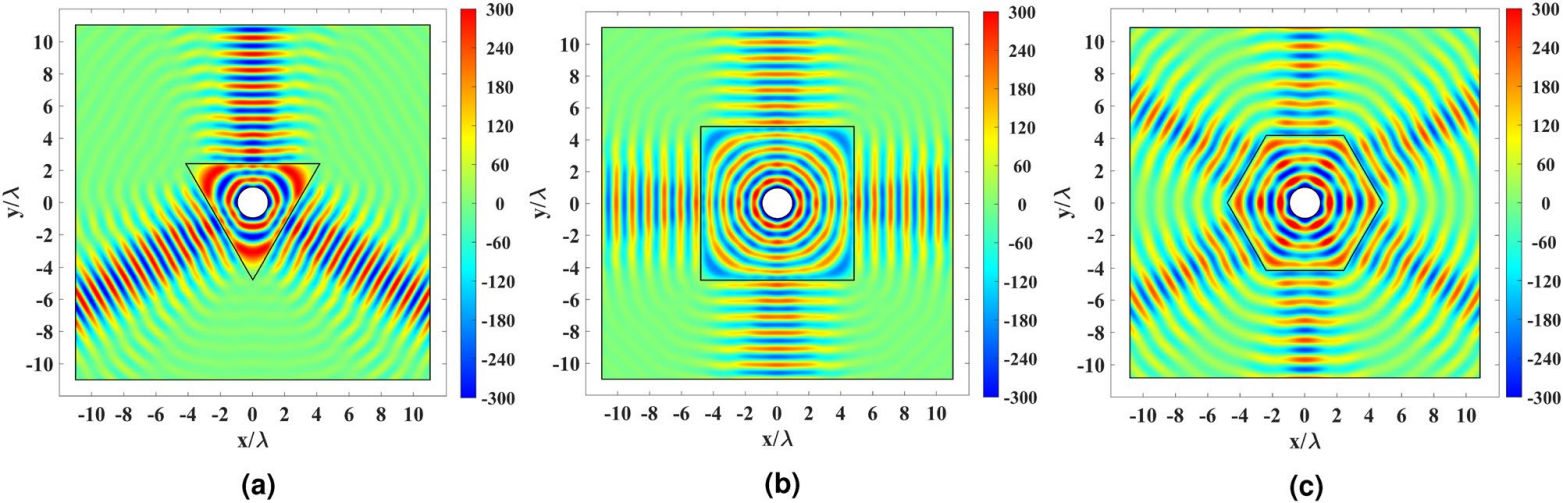
Real part of the electric field for the cylindrical wire excited by an electric current and enclosed by the physical space’s transformation medium. The results are shown for the transformation medium depicted in (**a**) Fig. 3b, (**b**) Fig. 3c, and (**c**) Fig. 3d.

For the full-wave simulation of the cases in Fig. 3b–d, an electrical current ${J_z}$ is defined on the circumference of the inner circle to produce the TE polarized cylindrical wave with the electric field component ${E_z}$. A perfectly matched layer (PML) is used around the simulation domain. The results inside the PML are curtailed for better illustration. The simulation results are represented in Fig. 8. The full-wave simulation results confirm the ray-tracing results in Fig. 7 as the transformation medium produces directive beams.

**Figure 9:**
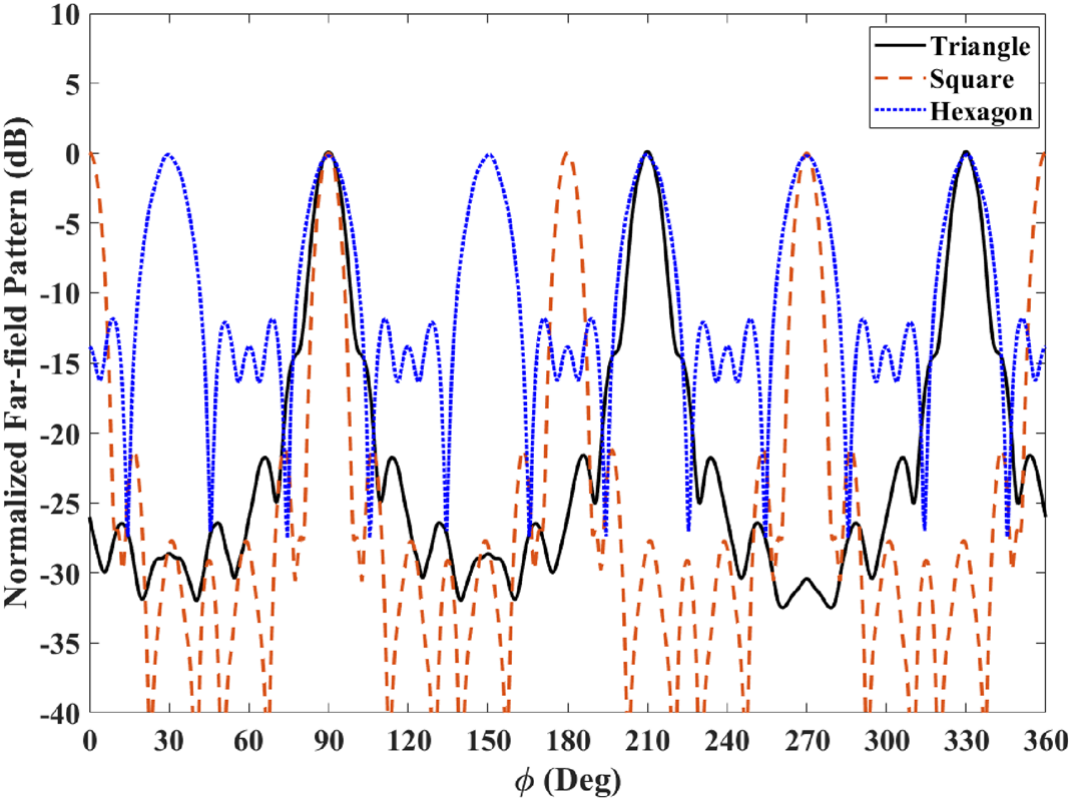
Normalized far-field pattern of the triangular, square, and hexagonal cases in Fig. 3b–d in dB.

To further investigate the device’s far-field performance, the normalized far-field patterns for the cases of Fig. 3b–d are plotted in decibels (dB) in Fig. 9. The triangular case has a higher half-power beamwidth (HPBW) than the square shape, even though it has the same aperture size. This is since a larger portion of its aperture contains a superluminal index. The square shape seems to possess lower HPBW values than the hexagon since it has a larger aperture, and it produces four beams instead of six beams.

**Figure 10:**
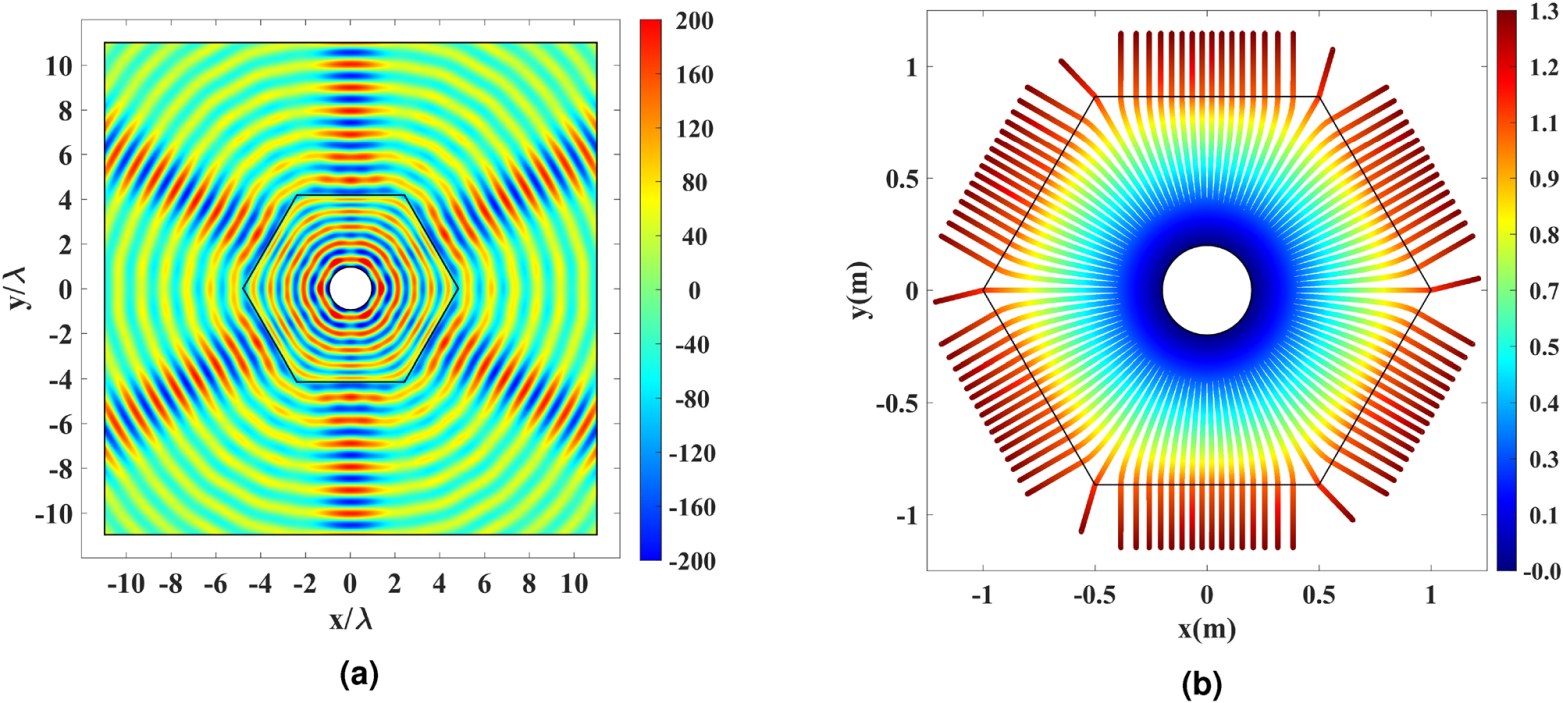
Simulation results for the rescaled refractive index of Fig. 5b. (**a**) Real part of the electric field for the cylindrical wire excited by an electric current. (**b**) Ray tracing results for 102 equally-spaced rays launched from the physical space’s inner rim. The color expression indicates the optical path length.

The full-wave and the ray-tracing simulation results are depicted in Fig. 10 for the rescaled hexagonal shell with the refractive index illustrated in Fig. 5b. 102 equally-spaced rays are launched from the physical space’s inner rim for the ray-tracing simulation. It is seen from both the full-wave and ray-tracing results that the directivity enhancing property is preserved after rescaling the optical path.

**Figure 11:**
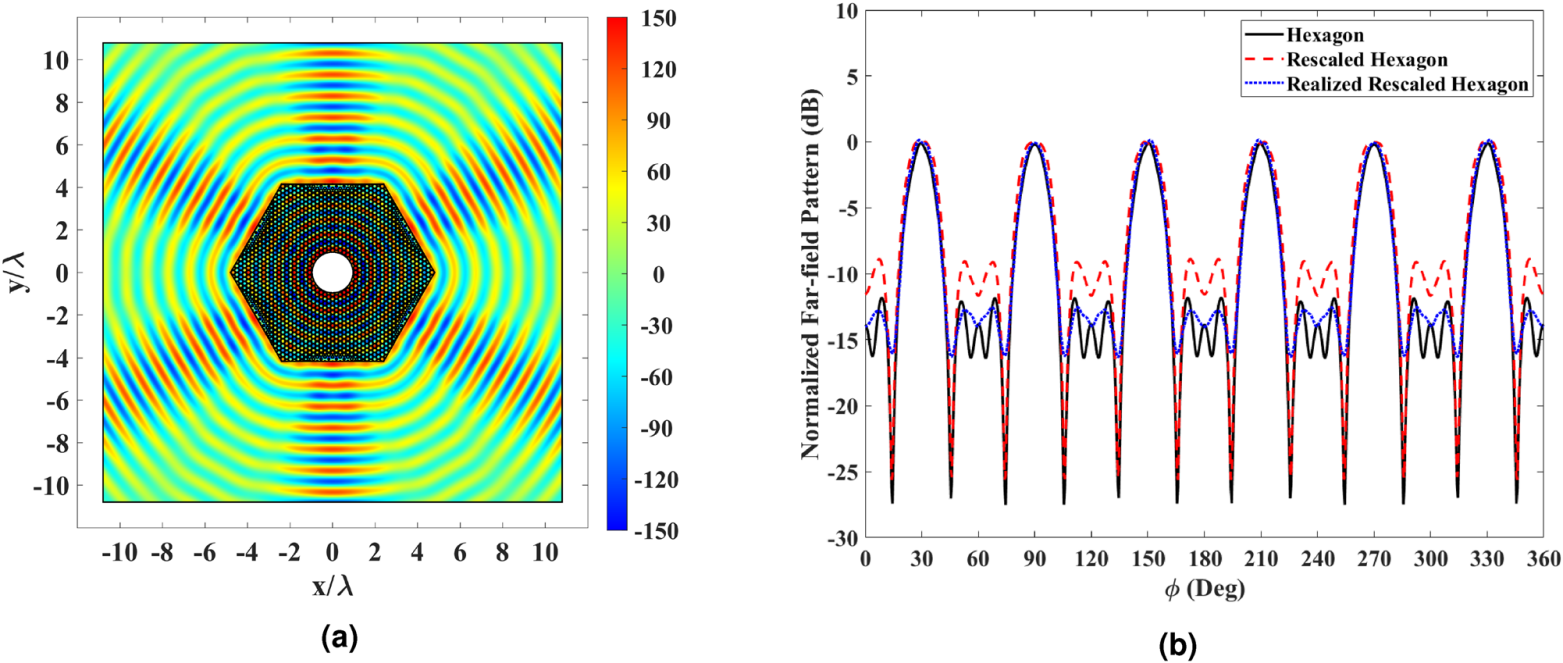
(**a**) Real part of the electric field for the cylindrical wire excited by an electric current and enclosed by the perforated structure in Fig. 6b. (**b**) Normalized far-field pattern of the hexagon in Fig. 3b, the rescaled hexagon in Fig. 5b, and the realized rescaled hexagon in Fig. 6b in dB.

The perforated AD600 structure in Fig. 6b has been simulated in the last simulation. Full-wave results are depicted in Fig. 11. It is seen that the realized hexagonal shell performs well and creates six directive beams. The far-field results are plotted for the dielectric shells of the hexagon (Fig. 3d), the rescaled hexagon (Fig. 5b), and the realized rescaled hexagon (Fig. 6b) to investigate the effects of optical path rescaling and realization. The hexagon and realized rescaled hexagon cases perform better in terms of HPBW and SLL.

## Conclusion

In conclusion, a method for designing a transformation medium has been proposed that can enhance the directivity of a cylindrical wire antenna. A nonhomogeneous dielectric shell is produced by establishing a strictly conformal map between an annulus and a doubly connected region. The doubly connected region consists of an inner cylindrical wire surrounded by an outer polygon. The device gradually flattens the phase fronts emanating from the wire as they reach the boundaries of the shell, leading to multiple directive beams. The effectiveness of the design method is demonstrated by designing and simulating three cases with triangular, square, and hexagonal outer polygons. The superluminal refractive index is remedied using the optical path rescaling method. The realization of the hexagonal case is done using the effective medium theory by perforating an Arlon AD600 substrate. The performance of all designs is verified by conducting full-wave and ray-tracing simulations.
